# Selection of plant oil as a supplemental energy source by monitoring rumen profiles and its dietary application in Thai crossbred beef cattle

**DOI:** 10.5713/ajas.18.0946

**Published:** 2019-02-14

**Authors:** Keiji Matsuba, Apirada Padlom, Anchalee Khongpradit, Phoompong Boonsaen, Prayad Thirawong, Suriya Sawanon, Yutaka Suzuki, Satoshi Koike, Yasuo Kobayashi

**Affiliations:** 1 Graduate School of Agriculture, Hokkaido University, Sapporo 060-8589, Japan; 2 Faculty of Agriculture at Kamphaeng Saen, Kasetsart University, Kamphaeng Saen, Nakhon Pathom, 73140, Thailand

**Keywords:** Blood Cholesterol, Feed Conversion Ratio, Palm Oil, Rumen Fermentation, Rumen Microbes

## Abstract

**Objective:**

The present study was conducted to select a plant oil without inhibitory effects on rumen fermentation and microbes, and to determine the optimal supplementation level of the selected oil in a series of *in vitro* studies for dietary application. Then, the selected oil was evaluated in a feeding study using Thai crossbred beef cattle by monitoring growth, carcass, blood and rumen characteristics.

**Methods:**

Rumen fluid was incubated with substrates containing one of three different types of plant oil (coconut oil, palm oil, and soybean oil) widely available in Thailand. The effects of each oil on rumen fermentation and microbes were monitored and the oil without a negative influence on rumen parameters was selected. Then, the dose-response of rumen parameters to various levels of the selected palm oil was monitored to determine a suitable supplementation level. Finally, an 8-month feeding experiment with the diet supplemented with palm oil was carried out using 12 Thai crossbred beef cattle to monitor growth, carcass, rumen and blood profiles.

**Results:**

Batch culture studies revealed that coconut and soybean oils inhibited the most potent rumen cellulolytic bacterium *Fibrobacter succinogenes*, while palm oil had no such negative effect on this and on rumen fermentation products at 5% or higher supplementation level. Cattle fed the diet supplemented with 2.5% palm oil showed improved feed conversion ratio (FCR) without any adverse effects on rumen fermentation. Palm oil-supplemented diet increased blood cholesterol levels, suggesting a higher energy status of the experimental cattle.

**Conclusion:**

Palm oil had no negative effects on rumen fermentation and microbes when supplemented at levels up to 5% *in vitro*. Thai crossbred cattle fed the palm oil-supplemented diet showed improved FCR without apparent changes of rumen and carcass characteristics, but with elevated blood cholesterol levels. Therefore, palm oil can be used as a beneficial energy source.

## INTRODUCTION

Kamphaeng Saen beef cattle were the first Thai beef cattle breed developed by Kasetsart University in order to produce high-quality beef under tropical conditions [1]. These cattle are a crossbreed between *Bos taurus* and *Bos indicus* (50% Charolais, 25% Brahman, and 25% Thai native), and are characterized by heat tolerance, tick resistance, higher growth and meat yield. In addition, another crossbreed between Kamphaeng Saen beef cattle and Japanese Black (Wagyu) has received considerable attention in terms of further improvements of meat quality.

To ensure high animal productivity and meat quality, beef cattle are often fed a high-concentrate starchy diet in developed countries. Such an intensive feeding system is becoming popular in Thailand as well, as the demand for beef, especially high-quality beef, has been increasing. However, feeding of a high-concentrate diet can cause rumen metabolic disorders such as acidosis, because large amounts of carbohydrate in the concentrate is rapidly degraded to produce lactate and short chain fatty acids (SCFA), thereby decreasing ruminal pH abnormally and hampering normal fermentation. This leads to lowered production efficiency in beef cattle [2–4]. Therefore, the use of an alternative feed energy source to rapidly fermentable carbohydrates might be an option.

Oil supplementation is one such alternative strategy of feed energy supply to beef cattle. As oil contains more than 2 times higher energy in comparison with carbohydrate or protein on a weight basis, oil is considered to be an efficient feed energy source. In addition, oil does not produce the same level of SCFA as starch. Therefore, oil supplementation is safer in terms of the risk of rumen acidosis. However, previous studies dealing with oil supplementation describe negative effects on rumen microbes, fiber digestion, fermentation, and dry matter intake (DMI) [5–7]. These adverse effects are remarkable especially when oil rich in unsaturated fatty acids is used [8]. Therefore, it is necessary to consider the type and supplementation level of oil suitable for normal rumen fermentation in beef cattle production.

This study was conducted to select a plant oil suitable for the use as a supplemental energy source, and to evaluate the selected oil in a feeding study with Thai crossbred beef cattle by monitoring growth, carcass, blood, and rumen characteristics.

## MATERIALS AND METHODS

All procedures in the present animal experiments were approved by the Animal Care and Welfare Committee of Hokkaido University, Japan, and were in accordance with the Animal Experiment Guidelines of Kasetsart University, Thailand.

### Batch culture study

A series of batch culture studies were carried out to select a suitable energy source from three different types of plant oil (coconut oil, palm oil, and soybean oil) and to determine the suitable dose level of the selected oil (palm oil, see Results). The three oils are widely available in Thailand, and were obtained from a feed company in Thailand (OLEEN Co. Ltd, Bangkok). Major fatty acids are lauric acid (46.7%), oleic acid (42.9%), and linoleic acid (54.6%) for coconut oil, palm oil, and soybean oil, respectively. Rumen contents were collected for batch culture studies using a stomach tube before feeding (08:00) from two Texel sheep fed once daily with timothy hay and a commercial concentrate (Mercian, Tokyo, Japan) diet at a 3:7 ratio (700 g in dry matter [DM]/d). The timothy hay contained 13% crude protein (CP), 50% neutral detergent fiber (NDF), and 60% total digestible nutrient (TDN), while the commercial concentrate contained 13% CP, 20% NDF and 76% TDN (all on a DM basis thereafter). Rumen contents were strained through two layers of surgical gauze and used for the following *in vitro* batch culture studies.

The first batch culture study was performed using three different types of plant oil to compare the effect of each oil supplementation on rumen fermentation and microbes. Artificial saliva [9] and the strained rumen fluid were combined at a 1:3 ratio (vol/vol). This mixture (10 mL) was dispensed as an inoculum to each test tube in which each oil and feed (timothy hay [0.06 g] and commercial concentrate [0.14 g]) had been placed. For this, each oil diluted with hexane was added to each tube at 5% of the total substrate. Tubes for control were prepared in the same manner but without the addition of oil. Tubes were left open overnight to allow the hexane to dissipate. The headspace of each tube after adding the inoculum was flushed with N_2_ gas and sealed with a butyl rubber stopper and plastic cap. Then, the tubes were incubated at 39°C for 24 h. Incubations were performed in five replicates. The second batch culture study was a dose-response assay using the palm oil selected in the first batch culture study (see Results), where six dose levels (0%, 2.5%, 5.0%, 7.5%, 10.0%, and 15% of total substrate) were tested in the same manner as above. After incubation, total gas production was measured through a needle-attached pressure gauge (Aϕ60B; GL Sciences, Tokyo, Japan), and gas samples were analyzed for H_2_, CH_4_, and CO_2_ using gas chromatography (see below). Cultures were used for pH, ammonia nitrogen, SCFA and microbial population analyses.

### Feeding study

Twelve Kamphaeng Saen steers (23 months old with an average body weight of 477.7±50.2 kg) were used. The feeding experiment consisted of 281 days (31-day adaptation period followed by 250-day experimental period). Animals were fed Napier grass hay (*ad libitum*), rice straw (2 kg/d), and concentrate (local formula) with or without palm oil supplementation twice daily (7:00 and 16:00). The Napier grass hay contained 7% CP, 0.7% ether extract (EE), and 66.5% NDF, and the rice straw contained 6.6% CP, 0.8% EE, and 68.3% NDF on a DM basis. Two different formulae of concentrate, with or without palm oil, were prepared; EE contents were higher in the concentrate supplemented with palm oil (0.9% vs 4.2%), but the other components were equalized (17.3% CP, 25.7% NDF). The main ingredients of the concentrate were cassava chips, soybean meal, palm kernel meal, molasses, vitamins, and minerals. The amount of concentrate fed was set at 1.25% of body weight during the adaptation period and the first half of the experimental period (1st to 4th month), and the amount was increased to 1.50% of body weight in the second half of the experimental period (5th to 8th month). All steers were fed the un-supplemented control concentrate during the adaptation period, and then seven steers were fed the same control concentrate, while another five steers were fed the concentrate supplemented with palm oil. The oil level was set at 2.5% of total feed. Leftover meals were measured daily, and body weights were measured monthly (1st to 4th month) or every two months (5th to 8th month) to obtain average daily gain (ADG), DMI, and feed conversion ratio (FCR). Jugular blood samples and rumen contents were collected from all steers at 4 h after morning feeding in the 4th, 6th, and 8th months of the experimental period. Blood serum was separated for biochemical analyses. Rumen contents obtained by a stomach tube were used for chemical and microbial analyses.

### Chemical analysis

Proximate chemical composition, NDF and acid detergent fiber of the experimental feeds was analyzed according to the method of the Association of Official Analytic Chemists [10]. Culture pH was measured using an electrode (pH METER F-51; HORIBA, Kyoto, Japan). Gases (H_2_, CH_4_, and CO_2_) in batch cultures were analyzed using a GC-8A gas chromatograph (Shimadzu, Kyoto, Japan) equipped with parallel columns of Porapak Q (Waters, Milford, MA, USA) and Molecular Sieve 13X (Restek, Bellefonte, PA, USA) and a thermal conductivity detector. SCFA was analyzed using a GC-14B gas chromatograph (Shimadzu, Japan) equipped with an ULBON HR-20M fused silica capillary column (0.53 mm i.d.× 30 m length, 3.0-μm film; Shinwa, Kyoto, Japan) and a flame-ionization detector. Injected samples were prepared as follows. Culture fluid or rumen fluid was mixed with 25% meta-phosphoric acid at a 5:1 ratio, incubated overnight at 4°C, and centrifuged at 10,000×g at 4°C for 10 min to obtain the supernatant, to which crotonic acid was added as an internal standard. The operational details of gas chromatography for gases and SCFA were as described by Watanabe et al [11]. Ammonia nitrogen was measured by the phenol-hypochlorite reaction method [12] using a microplate reader at 660 nm (ARVO MX, Perkin Elmer, Yokohama, Japan).

### Blood parameters

Total cholesterol, high density lipoprotein (HDL), low density lipoprotein (LDL), triglyceride, blood urea nitrogen (BUN), and glucose concentrations in blood samples were measured spectrophotometrically with an autoanalyzer (ARCHITECT *ci*8200; Abbott Laboratories, Chicago, IL, USA) using commercial kits (7D62 Cholesterol Reagent Kit, 3K33 Ultra HDL Reagent Kit, 1E31-20 MULTIGENT Direct LDL Reagent Kit, 7D74 Triglyceride Reagent Kit, 7D75 Urea Nitrogen Reagent Kit and 3L82-21 Glucose Reagent Kit; Abbott Laboratories, USA).

### Carcass and meat profiles

Steers were fasted for 24 h, weighed and slaughtered at a commercial abattoir. After slaughtering, carcasses were weighed and chilled at 4°C for 7 days. Then, carcasses on the right side were cut between the 12th and 13th rib and subjected to measurement of back fat thickness [13] and loin eye area according to Cacere et al [14]. The carcass pH was determined in *longissimus dorsi* muscles at 1, 48, and 168 h after slaughtering using a portable pH meter with a penetrating electrode probe (LE427, Mettler Toledo, Greifensee, Switzerland). The loin eye area was measured by tracing the outline onto tracing paper using an LI-3100 CArea Meter (LI-3100; Li-COR Biosciences, Lincoln, NE, USA). Then, three 2.5 cm thick steaks were removed from each rib section and trimmed of all external fat for measurement of drip loss [15], cooking loss [16], shear force with a Material Testing Machine (LR5K; Lloyd Instruments, West Sussex, UK), and marbling score by Thai Agricultural Commodity and Food Standard [17]. Meat color was measured using a color meter (HunterLab Mini Scan EZ, Reston, VA, USA) according to the L*, a*, b* system [18].

### Microbial analysis

DNA from the batch culture fluid and rumen contents in the feeding study was extracted and purified using the repeated bead beating plus column method [19] with a DNA stool mini kit (QIAamp DNA Stool Mini Kit; Qiagen, Hilden, Germany). The purified DNA was quantified using a NanoDrop 2000 (Thermo Fisher Scientific, Waltham, MA, USA) and diluted to 10 ng/μL before being subjected to polymerase chain reaction (PCR) amplification. The diluted DNA was used for quantitative real-time PCR (qPCR) to monitor the abundance of rumen representatives including total bacteria, total archaea, fungi, protozoa, *Fibrobacter succinogenes* (*F. succinogenes*), *Ruminococcus albus* (*R. albus*), *Ruminococcus flavefaciens* (*R. flavefaciens*), genus *Prevotella*, *Prevotella bryantii*, *Prevotella ruminicola* (*P. ruminicola*), *Anaerovibrio lipolytica* (*A. lipolytica*), and *Butyrivibrio* group. Details of qPCR such as primers, standards, PCR conditions, and calculations were as described by Koike et al [20] and Ohene-Adjei et al [21]. For protozoa and fungi, details were as found in Sylvester et al [22] and Denman and McSweeney [23], respectively. In brief, a standard plasmid containing the respective target gene sequence was obtained by PCR cloning using a target-specific primer set. The copy number of each standard plasmid was calculated using the molecular weight of nucleic acid and the length (base pair) of the cloned standard plasmid as described by Koike et al [20]. A LightCycler system and a KAPA SYBR Fast qPCR Kit (Kapa Biosystems, Charlestown, MA, USA) were used with 10-fold serial dilutions of standard plasmid for the respective target (16S rRNA gene or 18S rRNA gene sequence specific to each target microbe). Microbial quantity was estimated using amplification curves obtained from both the standard and sample. The specificity of PCR amplification was confirmed using melting curve analysis of the PCR products by increasing the temperature from 70°C to 95°C at a rate of 0.1°C/s. Microbial abundance was determined as absolute abundance of rRNA gene for total bacteria, protozoa, and fungi, or by relative proportion in total bacterial copy number for total archaea and specific bacteria.

### Statistical analysis

All data were shown as means with standard deviations. Results from batch culture studies were analyzed by one-way analysis of variance. Tukey’s honestly significant difference test was then conducted for multiple comparisons. Results from a feeding study were averaged for each treatment and compared (control vs palm oil) using Welch’s *t*-test. Statistical significance was considered at p<0.05 and a trend was defined at p<0.10.

## RESULTS

### Batch culture study for oil suitability

A comparison among three different types of oil (coconut oil, palm oil, and soybean oil) on their effects on rumen fermentation and microbial population is shown in Table 1. There were no significant differences in gas production, pH, SCFA concentrations and absolute abundance of total bacteria among the three different types of supplemented oil. Coconut oil decreased the molar proportion of acetate, while increasing that of butyrate and ammonia concentration. Coconut oil also decreased the absolute abundance of fungi and protozoa, the relative abundance of genus *Prevotella* and *F. succinogenes*, and increased that of *R. flavefaciens*, *R. albus* and total archaea. In particular, the decrease of *F. succinogenes* was drastic (0.62% vs 0.002% for control and coconut oil, respectively). In addition, the total sum of the relative abundance of predominant cellulolytic bacteria (*F. succinogenes*, *R. flavefaciens*, and *R. albus*) was also decreased by coconut oil supplementation (0.65% vs 0.11% for control and coconut oil, respectively). Soybean oil also decreased the relative abundance of *F. succinogenes*, though the decrease was not remarkable compared to coconut oil. In contrast, palm oil showed no change in any parameter when compared to the control. Based on these results, we selected palm oil as an energy source having no adverse effects on rumen fermentation and microbes.

### Batch culture study for dose-response to screened oil

The dose-response of ruminal fermentation and microbial population to supplementation of the selected palm oil is shown in Table 2. There were no significant differences in gas production, pH and ammonia concentration between the dose levels tested. Total SCFA concentration decreased as the dose level increased, showing a lower value at 15% supplementation compared to no supplementation. Acetate concentration decreased at 7.5% or higher level of palm oil supplementation. Propionate and butyrate concentrations also decreased at 15% supplementation. In molar proportion, propionate increased at 15% supplementation. The absolute abundance of total bacteria, protozoa and fungi did not show any differences between the dose levels tested. The relative abundance of *F. succinogenes* decreased with the dose level, showing a lower abundance at 7.5% or higher supplementation. The relative abundance of *A. lipolytica* increased at 15% supplementation. Meanwhile, the relative abundance of total archaea showed a lower value only at 10% supplementation. Other microbes did not show consistent changes within a range of palm oil supplementation of 0% to 15%.

### Feeding study with screened oil

Growth performance, and carcass and meat profiles of crossbred beef cattle fed the diet with or without palm oil are shown in Table 3. There were no differences in ADG and DMI between treatments. However, FCR tended to be improved in the first half of the experimental period (1st to 4th month) and improved significantly over the whole period (1st to 8th month). There was no difference in carcass profiles between treatments. Regarding meat profiles, pH (48 h after slaughtering) and CP content increased with palm oil feeding, while moisture content and marbling score tended to decrease. The EE content, cooking loss, drip loss, shear force and meat color did not show differences between treatments.

**Figure 1:**
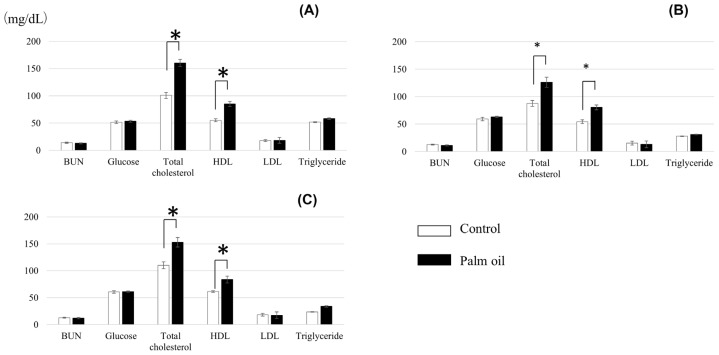
Blood parameters of crossbred cattle fed palm oil at 4th month (A), 6th month (B), and 8th month (C) of the experimental period (Mean values with standard deviations). * p<0.05.

Blood parameters of crossbred cattle fed the diet with or without palm oil are shown in Figure 1. There were no differences between treatments in concentrations of BUN, glucose, LDL, and triglyceride. Levels of total cholesterol and HDL were higher in cattle fed palm oil throughout the experimental period.

Rumen fermentation parameters of crossbred cattle fed palm oil are shown in Table 4. There were no differences in the concentration and molar proportion of SCFA, and ammonia concentration between treatments, except for the decreasing tendency in n-butyrate concentration at the 8th month. Though rumen pH tended to decrease at the 4th month during palm oil feeding, no other differences between treatments were observed at the other sampling periods.

Rumen microbial population of crossbred cattle fed the diet with or without palm oil supplementation is shown in Table 5. There were no differences in absolute abundance of total bacteria, protozoa and fungi between treatments. In relative abundance, palm oil feeding increased *P. ruminicola* and tended to increase *A. lipolytica* at the 4th month. At the 6th month, *Butyrivibrio* group increased and *P. ruminicola* tended to increase with palm oil feeding, while *R. albus* tended to decrease. The genus *Prevotella* and *A. lipolytica* increased with palm oil feeding at the 8th month.

## DISCUSSION

### Selection of oil as an energy source

The three types of oil tested in the present study (coconut oil, palm oil, and soybean oil) are widely available in Southeast Asia including Thailand, and are considered to be candidate oils for use as an energy source. These three types of oil have different fatty acid compositions, *e.g.*, coconut oil mainly consists of medium-chain fatty acids such as lauric acid (C12:0) [24], while palm oil and soybean oil are regarded as long-chain fatty acid sources, *e.g.*, palmitic acid (C16:0) and oleic acid (C18:1) for palm oil and linoleic acid (C18:2) for soybean oil [25].

Coconut oil drastically decreased the most potent cellulolytic bacterium *F. succinogenes* [26] (Table 1), which is in agreement with the previous study results showing decreases of *F. succinogenes in vitro* [27] and *in vivo* [28]. These decreases can be explained by bactericidal action of lauric acid, most major fatty acid of coconut oil, against rumen bacteria [24] that might be species-specific. Thus, coconut oil might depress fiber fermentation through this negative effect on *F. succinogenes*. Soybean oil also decreased *F. succinogenes* but to a lesser extent than coconut oil (Table 1). Meanwhile, we observed the increase of *R. flavefaciens* and *R. albus* with coconut oil supplementation (Table 1) as reported by previous studies [27,28], partially compensating for the decrease of *F. succinogenes*, as these three species are known as the predominant cellulolytic bacteria in the rumen [29]. However, this compensation is obviously not sufficient, since the total sum of these three species in the treatment is still lower than that in the control.

The decrease of protozoa by coconut oil supplementation (Table 1) was consistent with the results of previous reports [24,30,31]. As protozoa and cellulolytic bacteria produce acetate as a main fermentation product, the decrease of these microbes by coconut oil might have led to the reduction of the molar proportion of acetate (Table 1). The genus *Prevotella* is reported to be less sensitive to lauric acid [32] contained in coconut oil. Nevertheless, coconut oil decreased this genus remarkably in the present study (29.0%→14.3%, Table 1). This result suggests that coconut oil might inhibit unknown *Prevotella*, leading to the 50% reduction. As some phylogenetic groups of *Prevotella* are suggested to be involved in breaking down the hay diet [33], the decrease of *Prevotella* may be a negative sign for the maintenance of fiber fermentation.

Thus, we observed a negative influence of coconut oil on rumen microbes, especially those involved in fiber digestion, which may limit efficient feed utilization. Soybean oil also possesses a similar inhibitory influence but to a lesser extent. Meanwhile, no such negative action was observed for palm oil supplementation. Therefore, we selected palm oil as an energy source suitable for subsequent evaluations.

### Dose-response assay

We conducted a dose-response assay for palm oil to determine the supplemental level suitable for use in the feeding study. Total SCFA, propionate and butyrate concentrations decreased at 15% palm oil supplementation, and acetate concentration decreased at 7.5% or higher level (Table 2). These decreases indicate that rumen fermentation is likely inhibited by palm oil supplementation at high (≥7.5%) concentrations.

Propionate proportion increased at 15% supplementation of palm oil (Table 2), leading to the decrease of acetate to propionate (A/P) ratio. Previous *in vitro* and *in situ* studies report the increase of propionate proportion and decreases of acetate proportion and A/P ratio with supplementation of oleic acid [34] and canola oil rich in oleic acid [35,36], which are in good agreement with the present results using palm oil similarly rich in oleic acid.

The relative abundance of *F. succinogenes* decreased with the increase of palm oil supplementation, showing a significant decrease at 7.5% or higher level (Table 2). Zhang et al [34] reported that oleic acid, a main fatty acid of palm oil, did not inhibit the growth of *F. succinogenes* at a low supplementation level (3.5%), while growth was inhibited at a high supplementation level (7.0%). Therefore, the palm oil supplementation level must be appropriately set to avoid possible inhibitory effects on rumen fermentation, as the present dose-dependent change suggests.

These results, especially the decreases of acetate production and *F. succinogenes*, indicate that the level of palm oil supplementation might be limited up to 5%.

### Evaluation by feeding assay

A series of *in vitro* study results suggest that palm oil supplementation at 5% or lower level is suitable. Rumen fermentation, however, can be inhibited if the dietary fat content exceeds 6% to 7% [37]. Therefore, we employed 2.5% supplementation of palm oil in the total feed as a safer level in terms of rumen health and feed palatability.

Palm oil did not affect ADG and DMI (Table 3). Choi et al [38] fed a palm oil- or soybean oil-supplemented (3%) diet to Angus steers and found significant decreases of ADG and DMI for the soybean oil treatment, while no significant differences were observed for the palm oil treatment. Meanwhile, FCR in the present study tended to be improved in the first half of the experimental period and was improved significantly over the whole 8-month period (Table 3). This may be attributable to the improvement of energy intake level by palm oil supplementation. This speculation is supported by the higher serum levels of total cholesterol and HDL in cattle fed palm oil (Figure 1), reflecting their high status in energy intake, essentially led by higher EE level of palm oil-supplemented diet. Previous studies also report the increase in serum total cholesterol levels by oil supplementation to the feed [39–41].

The marbling score of meat tended to be decreased with palm oil feeding (Table 3), though the marbling scores were quite low in both treatments. Essentially, there was no significant difference in the carcass profiles determined, suggesting that palm oil feeding does not influence the meat quality.

Increases in genus *Prevotella* (8th month), *P. ruminicola* (4th and 6th months), and *A. lipolytica* (4th and 8th months) in the rumen of cattle fed palm oil may be due to their tolerance to the toxicity of oils and their component fatty acids [32,42]; however, these bacterial changes did not affect rumen fermentation profiles (SCFA and ammonia) throughout the experimental period (Table 4). Overall, as initially expected from the present *in vitro* evaluations, palm oil is assumed to serve as an energy source for experimental cattle without causing apparent changes in rumen fermentation.

## CONCLUSION

Palm oil supplementation improved FCR in Thai crossbreed beef cattle without any adverse effects on rumen fermentation and meat quality. Dose-response assays indicated no inhibition of rumen fermentation even at 5% palm oil supplementation in total substrate; thus, further study should be conducted with higher supplementation levels.

## Figures and Tables

**Table 1:** Comparison among 3 different types of fat (cocout oil, palm oil and soybean oil) influencing on rumen fermentation and microbial populations

Item	Control	Oil supplemented1)		

		Coconut	Palm	Soybean
Total gas (mL)	8.18±0.27	8.14±0.10	8.47±0.43	8.77±0.26
CO_2_ (mL)	6.75±0.22	6.69±0.09	7.01±0.37	7.27±0.25
CH_4_ (mL)	1.43±0.05	1.45±0.03	1.46±0.07	1.50±0.02
H_2_ (mL)	ND	ND	ND	ND
pH	6.10±0.07	6.07±0.05	6.04±0.07	6.13±0.02
Ammonia nitrogen (mg/dL)	3.5±0.4^b^	9.2±2.2^a^	4.5±1.0^b^	5.9±1.0^b^
Total SCFA (mmol/L)	93.2±9.3	87.7±4.6	92.3±3.8	91.8±3.0
Acetate (mmol/L)	45.5±4.3	42.1±2.2	44.9±2.0	44.4±1.4
Propionate (mmol/L)	31.7±3.2	29.1±1.9	30.9±1.2	31.1±1.0
n-Butyrate (mmol/L)	12.3±1.3	12.2±0.4	12.4±0.5	12.1±0.5
Acetate (molar %)	48.9±0.3^a^	48.0±0.2^b^	48.6±0.5^ab^	48.4±0.1^ab^
Propionate (molar %)	34.0±0.4	33.2±0.5	33.5±0.5	33.9±0.5
n-Butyrate (molar %)	13.2±0.2^b^	14.0±0.2^a^	13.4±0.04^b^	13.2±0.1^b^
Total bacteria (copies 16S rDNA/mL)	11.33±0.10	11.13±0.11	11.25±0.04	11.27±0.04
Protozoa (copies of 18S rDNA/mL)	9.70±0.09^a^	9.38±0.08^b^	9.63±0.06^a^	9.67±0.04^a^
Fungi (copies of 18S rDNA/mL)	7.90±0.02^a^	7.19±0.08^b^	8.05±0.15^a^	7.85±0.15^a^
Archaea (% of total bacteria)	0.48±0.07^b^	0.88±0.12^a^	0.49±0.07^b^	0.56±0.09^b^
Genus *Prevotella* (% of total bacteria)	29.01±3.22^a^	14.26±0.97^b^	25.70±2.78^a^	25.22±2.24^a^
*Butyrivibrio* group (% of total bacteria)	8.49±1.12	9.69±0.70	7.74±0.29	7.97±1.98
*Fibrobacter succinogenes* (% of total bacteria)	0.62±0.14^a^	0.002±0.0001^c^	0.56±0.08^ab^	0.36±0.07^b^
*Ruminococcus flavefaciens* (% of total bacteria)	0.026±0.021^b^	0.088±0.012^a^	0.059±0.010^ab^	0.031±0.009^b^
*Ruminococcus albus* (% of total bacteria)	0.008±0.002^b^	0.023±0.006^a^	0.017±0.004^ab^	0.009±0.002^b^
*Prevotella ruminicola* (% of total bacteria)	1.38±0.12	0.82±0.12	1.10±0.18	1.21±0.37
*Prevotella bryantii* (% of total bacteria)	ND	ND	ND	ND
*Anerovibrio lipolytica* (% of total bacteria)	0.076±0.010^ab^	0.112±0.020^a^	0.073±0.014^b^	0.071±0.006^b^

ND, not detected; SCFA, short chain fatty acid.

1) Each oil was supplemented at 5% of total substrate on a dry matter basis.

a–c Values in the same row with different script letters differ significantly (p<0.05).

**Table 2:** Dose response of ruminal fermentation and microbial population to palm oil supplementation

Item	Palm oil					

	0%	2.5	5%	7.5%	10%	15%
Total gas (mL)	11.32±0.07	11.27±0.55	11.39±0.37	10.85±1.07	11.43±0.37	10.44±1.66
CO_2_ (mL)	7.10±0.28	7.18±0.13	7.07±0.12	6.80±0.48	7.13±0.24	6.79±1.01
CH_4_ (mL)	4.22±0.27	4.09±0.43	4.32±0.32	4.06±0.65	4.30±0.18	3.64±0.67
H_2_ (mL)	ND	ND	ND	ND	ND	ND
pH	6.12±0.03	6.15±0.01	6.16±0.03	6.16±0.02	6.14±0.01	6.14±0.01
Ammonia nitrogen (mg/dL)	8.6±1.2	7.8±0.5	8.8±1.7	6.1±1.1	7.2±1.5	9.0±1.2
Total SCFA (mmol/L)	88.9±5.5^a^	88.4±2.4^a^	87.3±2.0^a^	81.2±1.3^a^	81.6±4.9^a^	70.3±4.6^b^
Acetate (mmol/L)	42.1±2.7^a^	42.2±1.1^a^	41.6±1.1^ab^	38.2±0.6^b^	38.2±2.4^b^	32.7±1.9^c^
Propionate (mmol/L)	27.8±1.9^a^	27.4±0.8^a^	26.9±0.4^a^	25.4±0.7^ab^	25.7±1.6^a^	22.7±1.9^b^
n-Butyrate (mmol/L)	15.3±0.8^a^	15.3±0.5^a^	15.3±0.4^a^	14.2±0.2^a^	14.3±0.8^a^	12.4±0.9^b^
Acetate (molar %)	47.4±0.4^ab^	47.7±0.5^a^	47.6±0.3^a^	47.1±0.4^ab^	46.7±0.2^b^	46.5±0.6^b^
Propionate (molar %)	31.3±0.3^b^	31.0±0.3^b^	30.9±0.3^b^	31.3±0.4^ab^	31.5±0.4^ab^	32.2±0.7^a^
n-Butyrate (molar %)	17.2±0.3	17.3±0.3	17.6±0.2	17.5±0.1	17.6±0.2	17.7±0.3
Total bacteria (copies 16S rDNA/mL)	11.94±0.07	11.90±0.11	11.89±0.02	11.97±0.09	12.03±0.07	11.97±0.07
Protozoa (copies of 18S rDNA/mL)	10.18±0.03	10.18±0.08	10.19±0.06	10.27±0.07	10.32±0.03	10.28±0.02
Fungi (copies of 18S rDNA/mL)	7.80±0.10	7.89±0.12	7.80±0.28	7.84±0.39	8.00±0.17	8.29±0.19
Archaea (% of total bacteria)	5.24±0.35^a^	4.67±1.14^ab^	5.23±0.71^a^	5.13±0.81^a^	3.03±0.12^b^	3.86±0.76^ab^
Genus *Prevotella* (% of total bacteria)	18.63±0.78^ab^	16.05±1.44^b^	20.50±0.88^a^	19.00±1.35^ab^	16.38±1.79^b^	16.29±0.69^b^
*Butyrivibrio* group (% of total bacteria)	9.54±0.68	8.06±1.36	8.54±0.37	9.01±0.35	8.20±0.91	9.72±0.84
*Fibrobacter succinogenes* (% of total bacteria)	0.37±0.04^a^	0.33±0.09^ab^	0.27±0.02abc	0.19±0.05^bc^	0.20±0.03^c^	0.14±0.01^c^
*Ruminococcus flavefaciens* (% of total bacteria)	0.025±0.003	0.029±0.001	0.034±0.006	0.034±0.003	0.031±0.002	0.030±0.004
*Ruminococcus albus* (% of total bacteria)	0.0067±0.0019	0.0096±0.0012	0.0069±0.0015	0.0102±0.0009	0.0077±0.0007	0.0099±0.0031
*Prevotella ruminicola* (% of total bacteria)	0.40±0.03^ab^	0.34±0.05^b^	0.46±0.05^a^	0.44±0.02^ab^	0.33±0.05^b^	0.39±0.05^ab^
*Prevotella bryantii* (% of total bacteria)	ND	ND	ND	ND	ND	ND
*Anerovibrio lipolytica* (% of total bacteria)	0.090±0.006^b^	0.093±0.016^b^	0.096±0.002^b^	0.119±0.010^b^	0.110±0.011^b^	0.159±0.013^a^

ND, not detected; SCFA, short chain fatty acid.

a–c Values in the same row with different script letters differ significantly (p<0.05).

**Table 3:** Growth performance, carcass and meat profiles of crossbred cattle fed palm oil

Item		Control	Palm oil
Growth performance			
Initial weight (kg)		486.6±68.4	481±42.5
Final weight (kg)		649.1±88.7	663.8±63.1
Average daily gain (kg/d)	1–4 mo	0.62±0.17	0.70±0.08
	5–8 mo	0.68±0.08	0.76±0.12
	1–8 mo	0.65±0.10	0.73±0.09
Dry matter intake (kg/d)	1–4 mo	8.6±1.2	8.5±1.0
	5–8 mo	8.6±1.1	8.5±1.0
	1–8 mo	8.6±1.2	8.5±1.0
Feed conversion ratio	1–4 mo	14.3±2.9	12.1±0.6**
	5–8 mo	12.8±0.8	11.4±1.6
	1–8 mo	13.3±0.8	11.7±1.1*
Carcass profile			
Live weight (kg)		616.1±90.9	632.6±67.2
Carcass weight (kg)		377.6±112.3	382.4±88.3
Loin eye area (cm^2^)		85.5±12.5	87.7±9.7
Back fat thickness (cm)		1.3±0.8	1.2±0.5
Meat profile			
pH	1 h	6.72±0.13	6.76±0.07
	48 h	5.65±0.02	5.69±0.02*
	168 h	5.49±0.06	5.53±0.04
Moisture (%)		68.1±2.2	65.4±2.5**
Crude protein (%)		23.5±1.9	25.8±1.2*
Ether extract (%)		7.2±1.3	8.1±2.3
Drip loss (%)		3.6±0.8	2.9±0.4
Cooking loss (%)		26.4±4.9	25.0±4.4
Shear force (kgf)		4.7±1.6	4.6±1.0
Marbling score		1.6±0.3	1.3±0.2**
Meat color index	L*	39.8±1.7	38.4±1.6
	a*	19.0±0.5	19.4±0.6
	b*	16.2±0.8	15.7±0.9

** p<0.10,

* p<0.05.

**Table 4:** Rumen fermentation parameters of crossbred cattle fed palm oil

Item	Control	Palm oil
4th month		
pH	6.60±0.27	6.17±0.36**
Ammonia nitrogen (mg/dL)	11.4±5.3	9.5±5.8
Total SCFA (mmol/L)	97.4±32.3	103.0±9.3
Acetate (mmol/L)	62.3±17.7	65.2±5.1
Propionate (mmol/L)	16.4±7.7	18.1±2.1
n-Butyrate (mmol/L)	15.4±6.6	16.1±2.5
Acetate (molar %)	64.6±3.9	63.3±1.3
Propionate (molar %)	16.4±2.5	17.5±0.9
n-Butyrate (molar %)	15.5±2.1	15.6±1.6
6th month		
pH	6.47±0.41	6.46±0.37
Ammonia nitrogen (mg/dL)	15.5±3.3	14.0±6.6
Total SCFA (mmol/L)	102.5±17.5	93.4±17.8
Acetate (mmol/L)	60.5±6.9	52.7±10.9
Propionate (mmol/L)	20.9±9.9	20.0±4.3
n-Butyrate (mmol/L)	16.5±3.2	15.6±4.2
Acetate (molar %)	59.6±4.5	56.3±2.1
Propionate (molar %)	19.8±6.0	21.7±4.8
n-Butyrate (molar %)	16.1±2.1	16.6±2.4
8th month		
pH	6.52±0.21	6.61±0.25
Ammonia nitrogen (mg/dL)	11.3±3.4	14.7±5.0
Total SCFA (mmol/L)	88.8±20.2	74.3±11.7
Acetate (mmol/L)	56.9±15.6	46.6±6.9
Propionate (mmol/L)	14.6±2.6	13.9±3.8
n-Butyrate (mmol/L)	13.6±2.5	10.9±1.8**
Acetate (molar %)	63.6±3.4	62.9±3.3
Propionate (molar %)	16.7±2.2	18.5±2.7
n-Butyrate (molar %)	15.4±1.55	14.7±1.1

SCFA, short chain fatty acid.

** p<0.10.

**Table 5:** Rumen microbial population of crossbred cattle fed palm oil

Item	Control	Palm oil
4th month		
Total bacteria (copies 16S rDNA/mL)	11.68±0.32	11.46±0.24
Protozoa (copies of 18S rDNA/mL)	10.35±0.50	10.14±0.34
Fungi (copies of 18S rDNA/mL)	7.67±0.60	7.12±0.54
Archaea (% of total bacteria)	2.70±2.19	3.54±3.93
Genus *Prevotella* (% of total bacteria)	18.07±5.05	29.64±12.76
*Butyrivibrio* group (% of total bacteria)	3.06±0.85	2.84±0.87
*Fibrobacter succinogenes* (% of total bacteria)	0.16±0.09	0.10±0.12
*Ruminococcus flavefaciens* (% of total bacteria)	0.06±0.04	0.08±0.05
*Ruminococcus albus* (% of total bacteria)	0.014±0.009	0.009±0.007
*Prevotella ruminicola* (% of total bacteria)	0.13±0.08	0.34±0.11*
*Prevotella bryantii* (% of total bacteria)	0.00001±0.00002	0.00014±0.00016
*Anerovibrio lipolytica* (% of total bacteria)	0.008±0.011	0.026±0.017**
6th month		
Total bacteria (copies 16S rDNA/mL)	11.31±0.23	11.28±0.17
Protozoa (copies of 18S rDNA/mL)	10.13±0.41	10.05±0.34
Fungi (copies of 18S rDNA/mL)	6.75±1.64	6.45±1.37
Archaea (% of total bacteria)	1.93±1.83	3.20±2.94
Genus *Prevotella* (% of total bacteria)	26.97±15.36	23.48±8.73
*Butyrivibrio* group (% of total bacteria)	2.39±0.56	3.50±0.88*
*Fibrobacter succinogenes* (% of total bacteria)	0.10±0.10	0.06±0.07
*Ruminococcus flavefaciens* (% of total bacteria)	0.104±0.077	0.098±0.055
*Ruminococcus albus* (% of total bacteria)	0.013±0.011	0.004±0.002**
*Prevotella ruminicola* (% of total bacteria)	0.17±0.11	0.28±0.10**
*Prevotella bryantii* (% of total bacteria)	0.015±0.033	0.014±0.008
*Anerovibrio lipolytica* (% of total bacteria)	0.025±0.019	0.052±0.037
8th month		
Total bacteria (copies 16S rDNA/mL)	11.59±0.20	11.56±0.16
Protozoa (copies of 18S rDNA/mL)	10.64±0.12	10.61±0.15
Fungi (copies of 18S rDNA/mL)	7.91±0.41	7.92±0.17
Archaea (% of total bacteria)	1.86±0.99	1.54±0.87
Genus *Prevotella* (% of total bacteria)	18.44±3.28	24.60±3.20*
*Butyrivibrio* group (% of total bacteria)	2.35±0.83	2.23±0.45
*Fibrobacter succinogenes* (% of total bacteria)	0.25±0.16	0.23±0.18
*Ruminococcus flavefaciens* (% of total bacteria)	0.22±0.08	0.20±0.09
*Ruminococcus albus* (% of total bacteria)	0.02±0.011	0.01±0.003
*Prevotella ruminicola* (% of total bacteria)	0.15±0.08	0.23±0.13
*Prevotella bryantii* (% of total bacteria)	0.001±0.001	0.003±0.003
*Anerovibrio lipolytica* (% of total bacteria)	0.009±0.005	0.018±0.005*

** p<0.10,

* p<0.05.
